# NKP30-B7-H6 Interaction Aggravates Hepatocyte Damage through Up-Regulation of Interleukin-32 Expression in Hepatitis B Virus-Related Acute-On-Chronic Liver Failure

**DOI:** 10.1371/journal.pone.0134568

**Published:** 2015-08-04

**Authors:** Yong Zou, Junjie Bao, Xingfei Pan, Ying Lu, Sihong Liao, Xicheng Wang, Guoying Wang, Dongjun Lin

**Affiliations:** 1 Department of Blood Transfusion, the Third Affiliated Hospital of Sun Yat-Sen University, Guangzhou, China; 2 Preterm Birth Prevention and Treatment Research Unit, Department of Obstetrics, Guangzhou Women and Children's Medical Center, Guangzhou, China; 3 Department of infectious disease, the Third Affiliated Hospital of Guangzhou Medical University, Guangzhou, China; 4 Department of oncology, Zhangqiu People’s Hospital, Jinan, China; 5 Department of Liver Transplantation Center, the Third Affiliated Hospital of Sun Yat-Sen University, Guangzhou, China; 6 Department of Hematology, the Third Affiliated Hospital of Sun Yat-Sen University, Guangzhou, China; University of Sydney, AUSTRALIA

## Abstract

**Background and Aims:**

Previous work conducted by our group has shown that the accumulation of hepatic natural killer (NK) cells and the up-regulation of natural cytotoxicity receptors (NKP30 and NKP46) on NK cells from patients with hepatitis B virus-related acute-on-chronic liver failure (HBV-ACLF) were correlated with disease progression in HBV-ACLF. The natural cytotoxicity receptors expressed on NK cells are believed to be probable candidates involved in the NK cell-mediated hepatocyte damage in HBV-ACLF. However, the underlying mechanisms remain to be elucidated. In the present study, we aimed to discover the role of NKP30-B7-H6 interaction in NK cells-mediated hepatocyte damage in HBV-ACLF.

**Methods:**

Hepatic expressions of B7-H6 and interleukin-32 (IL-32) were examined by immunochemistry staining in samples from patients with HBV-ACLF or mild chronic hepatitis B (CHB). The cytotoxicity of NK-92 cell against target cells (Huh-7 and LO2) was evaluated by CCK8 assay. Expression of IL-32 in liver NK cell, T cells and NK-92 cell line was detected by the flow cytometric analysis. The effect of IL-32 on the apoptosis of Huh7 cells was evaluated using Annexin V/PI staining analysis.

**Results:**

An enhancement of hepatic B7-H6 and IL-32 expression was associated with the severity of liver injury in HBV-ACLF. And there was a positive association between hepatic B7-H6 and IL-32 expression. Expressions of IL-32 in liver NK cells and T cells were increased in HBV-ACLF patients. In vitro NK-92 cells are highly capable of killing the high B7-H6 expressing Huh7 cells and B7-H6-tansfected hepatocyte line LO2 cells dependent on NKP30 and B7-H6 interaction. Furthermore, NK-92 cells exhibited elevated IL-32 expression when stimulated with anti-NKP30 antibodies or when co-cultured with Huh7 cells. IL-32 can induce the apoptosis of Huh7 cells in a dose-dependent manner.

**Conclusion:**

Our results suggest that NKP30-B7-H6 interaction can aggravate hepatocyte damage, probably through up-regulation of IL-32 expression in HBV-ACLF.

## Introduction

Hepatitis B virus-related acute-on-chronic liver failure (HBV-ACLF) is the most common severe diseases requiring immediate hospitalization in China and many other Asian countries [1–5]. A characteristic of this disease is the extreme rapidity of the necromicroinflammatory process, resulting in widespread or complete hepatocellular necrosis in weeks or even days [6]. Although multiple factors have been implicated in disease development, it is generally accepted that immune cells-mediated liver injury play a critical role [7–9]. Our previous study found that NK cells were recruited dramatically in the livers of patients with HBV-ACLF. In addition, expression of the natural cytotoxicity receptors (NKp30 and NKp46) on the peripheral NK cells was unregulated in patients with HBV-ACLF [10]. These findings suggested an important role of NK cells in the pathogenesis HBV-ACLF.

Accumulating evidence has shown that the natural cytotoxicity receptors expressed on NK cells play a dominant role in NK cell activation during the process of natural cytotoxicity against tumor cells and virus-infected target cells. The natural cytotoxicity receptors are also considered potential candidates involved in NK cell-mediated hepatocyte damage in HBV-ACLF. However, the underlying mechanisms remain unclear. In the current study, we reported that the NKp30 ligand B7-H6 and the proinflammatory cytokine IL-32 were both highly up-regulated in the livers of patients with HBV-ACLF and that their expression levels were highly positively correlated with the severity of liver injury. Furthermore, *in vitro* cytotoxicity assay demonstrated that NKP30-B7-H6 interaction unregulated IL-32 expression and induced hepatoma cells apoptosis.

## Materials and Methods

### Study Subjects

The research protocol was reviewed and approved by the institutional review board of the Third Hospital of Sun Yat-Sen University, Guangzhou, People’s Republic of China. We enrolled thirty patients with HBV-ACLF and thirty mild CHB patients in this study and informed written consent was obtained from each patients. Needle biopsy liver tissues were obtained from patients with mild CHB at the department of infectious disease, the Third Hospital of Sun Yat-Sen University. Resected liver tissue samples were obtained from HBV-ACLF patients who underwent liver transplant at the liver transplant center, the Third Hospital of Sun Yat-Sen University. Biochemical, histological and clinical features were used for the diagnoses of mild CHB and HBV-ACLF. ACLF was diagnosed according to the criteria established by the Asian Pacific Association for the study of the liver (APASL) about ACLF [11]. Individuals with concurrent HCV, hepatitis D virus, hepatitis G virus, HIV infections and autoimmune liver diseases were excluded. The clinical characteristics of all patients in this study are shown in Table 1 and S1 Table.

**Table 1:** Characteristics of the patients (Immunochemistry staining).

Characteristic	Mild CHB	HBV-ACLF
Number of patients	20	20
Age (years)	32(20–30.5)	39(34.5–45)
Male gender (n%)	14(70.0%)	15(75%)
HBsAg+ (%)	100	65
HBeAg+ (%)	40	20
Anti-HBcAg+ (%)	100	100
HBV DNA log_10_ (copies/ml)*	5.9(3.6–8.3)	3.7(3.0–5.9)
Serum ALT (IU/L)	112(66–223.5)	116.5(64.0–245.5)
Serum total bilirubin (μmol/L)*	14.4(11.7–11.6)	596.0(440.1.3–843.7)
Prothronbinase time (PT)*	12.0(11.5–12.7)	32.3(27.5–39.5)
Prothrombinase activity (PTA)*	103.0(96.0–112.3)	23.4(20.0–27.3)

* Median (interquartile range)

### Cells Culture

Cell lines including NK-92, Huh7, HepG2, HepG2.215, LO2, K562 and HeLa were all obtained from the American Type Culture Collection (ATCC, Manassas, VA, USA).The NK-92 cell line was cultured in Minimum Essential Medium Alpha (MEM-α) Medium (Invitrogen) containing 12.5% horse serum (Invitrogen),12.5% fetal bovine serum (FBS) (Invitrogen), and 100 IU/ml recombinant human interleukin 2 (IL-2) (Peprotech, Rocky Hill, NJ, USA).The cell line LO2 and K562 were cultured in RPMI-1640 medium (Invitrogen) containing 10% FBS. The cell lines Huh7, HepG2 and HepG2.215 were cultured in Dulbecco’s modified Eagle’s media (DMEM) (Invitrogen) containing 10% FBS.

### Immunohistochemical Staining of B7-H6 and IL-32

Paraffin-embedded liver tissue samples obtained from patients with mild CHB or liver transplant patients with HBV-ACLF were used. The sections (4 μm) were treated with 0.03% H_2_O_2_/NaNO_3_ to block endogenous peroxidase, treated with 10% normal goat serum (30 min) to block nonspecific staining, incubated with an antibody against IL-32 (Catalog # 513402, BioLegend, San Diego, CA) or B7-H6 (Catalog # ab121796, Abcam, USA) or with rabbit serum as a negative control at 4°C overnight in a humid chamber, washed in PBS, incubated with HRP-conjugated secondary antibody (Catalog # GK500705, DAKO, Kyoto, Japan) for 30 min, and visualized after staining with 3,3,2-diaminobenzidine (DAB) solution (DAKO, Kyoto, Japan), followed by counterstaining with a hematoxylin solution. The sections were analyzed under a light microscope.

### Image analysis

Immunohistochemical images were captured digitally. The mean density (integrated optical density sum/ positive area sum) of all of the diaminobenzidine-stained areas in each photo was measured to determine IL-32 and B7-H6 expression. All analyses were performed using Image-Pro Plus 6.0 software (Diagnostic Instruments, Sydney, NSW, Australia). Data were expressed as relative mean density, which was determined by computer-aided planimetry. Twenty images per sample were analyzed to identify the mean density of all of the diaminobenzidine-stained areas in each photo.

### Liver mononuclear cells (MNCs) isolation

Liver biopsy and resected liver tissues were dissected, pressed through a 200-gauge stainless steel mesh, suspended in RPMI 1640 medium containing 5% FBS, centrifuged at 30g for 3 min. The suspension was transferred and centrifuged at 500g for 10 min. The pellet was resuspended in 40% Percoll (GE Healthcare, Uppsala, Sweden) solution in RPMI 1640 medium, layered on to 70% Percoll solution, and centrifuged for 20 min at 800g. The liver mononuclear cells (MNCs) were collected from the interphase. The cells were washed and resuspended with PBS containing 0.1% BSA and 0.01% sodium azide (FACS buffer).

### Flow cytometric analysis

To analyze the expression of NK cell receptors on NK-92 cells, the NK-92 cells were stained with the following antibodies: PE-conjugated anti-NKP30 (Catalog # 558407, BD Biosciences), PE-conjugated anti-NKP46 (Catalog # 557991, BD Biosciences), PE-conjugated anti-CD158a (Catalog # 556063, BD Biosciences), PE-conjugated anti-NKG2A (Catalog # FAB1059P, R&D Systems), PE-conjugated anti-NKG2D (Catalog # FAB139P, R&D Systems), PE-conjugated lgG1 isotype control (Catalog # 556063, BD Biosciences), and PE-conjugated lgG2A isotype control (Catalog # IC003P, R&D Systems). Intracellular cytokine staining analysis was used to detect the expression of IL-32 in NK-92 cells, liver NK cells and T cells. In brief, NK-92 cells were stimulated by directly adding an anti-NKP30 monoclonal antibody (Catalog # MAB18491, Clone 210847, R&D Systems) and IgG2A Isotype Control (Catalog # 20102, R&D Systems) with final concentration of 1ug/ml or co-culturing with Huh7 cells in the presence of BD GolgiStop Protein Transport inhibitor. Liver NK cells and T cells were stimulated by phorbol myristate acetate (PMA) and inomycin in the presence of BD GolgiStop Protein Transport inhibitor. After stimulation for 4h, cells were washed, fixed, permeabilized and stained with APC-conjugated anti-IL-32 (Catalog # IC30402A, R&D Systems) and APC-conjugated lgG2A isotype control (Catalog # IC006A, R&D Systems).

### Construction of recombinant plasmid pIRES2-EGFP-B7-H6

The cDNA of human B7-H6 was purchased from GeneCopoeia. Constructs of the recombinant overexpression plasmid pIRES2-EGFP-B7-H6 was generated by subcloning the B7-H6 cDNAs into pIRES2-EGFP vector (BD Clontech, USA).

The recombinant plasmid was transfected into LO2 cells using lipofectamin 2000 (Invitrogen, USA) according to the manufacturer's protocol. B7-H6 expression was detected under fluorescence microscope and confirmed by quantitative real time PCR and western blotting.

### siRNA-mediated knockdown

A chemically synthesized B7-H6 siRNA (Guangzhou RiboBio Co., Ltd., Guangzhou, Guangdong, China) is a pool of 3 target-specific 20–25 nt siRNA designed to knock down B7-H6 gene expression in Huh7 cells. Ascramble siRNA duplex was also generated to serve as a non-target control. Lipofectamine 2000 transfection reagent (Invitrogen Co., Carlsbad, CA, USA) was used for transfection following the manufacturer's instructions. B7-H6 expression after siRNA knockdown was assessed by quantitative real time PCR and western blotting.

### Cytotoxicity assay

The CCK8 (cell counting kit 8) assay was used to evaluate the cytotoxic activities of NK-92 cells.Huh-7 cells transfected with or no B7-H6 siRNA and LO2 cells transfected with or no pIRES2-EGFP-B7-H6 plamid were used target cells. Target cells were seeded in 96-well plates, with 1×10^4^ cells in each well. The NK-92 effector cells were incubated with target cells at effector/target ratios of 1:1, 1:5, 1:10 or 1:25 for 4 h. A 15 μL aliquot of CCK8 solution (5 mg/ml in PBS) was then added to each well, and the plates were incubated at 37°C for 4 h. The optical density (OD) in each well was measured at 540 and 630 nm. The percentage of cytolysis was calculated as{[T OD value—(E with T OD value)]/T OD value}×100, where T = target cells, E = effector cells, E with T = effector cells cultured together with target cells and OD = optical density. Soluble NKp30-Fc protein (Catalog # 1849-NK, 2ug/ml, R&D Systems) and control IgG1 Fc (Catalog # 110-HG, 2ug/ml, R&D Systems) were used for the blocking assay.

### Cell apoptosis assay

Huh7 cells were treated with various concentration of recombinant Human IL-32γ (Catalog # 4690-IL/CF, 0–0.4μg/ml, R&D Systems) for 48h, and collected by trypsinization and centrifugation at 1500 rpm for 5 minutes, followed by washing cell pellet twice with cold PBS and resuspending cell pellet in Annexin-binding buffer. Then, cells were added 5 μL Annexin V and 1 μL 100 μg/ml PI working solution in 100 μL cell suspension, and incubated at room temperature for 15 minutes in the dark. After the incubation, 400 μL Annexin-binding buffer was added. After mixing gently, the fluorescence intensity was detected with the flow cytometry. The percentage of cells stained by Annexin V/PI which indicates early apoptosis was shown in bar chart.

### Real-time PCR assays

B7-H6 mRNA expression was assessed in Huh7, HepG2, HepG2.215, LO2, K562, and HeLa cells by quantitative real-time PCR. The details were as follows: Total RNA was extracted from cell samples using TRIZOL reagent (Invitrogen) according to the manufacturer’s instructions. Subsequently, Total RNA (1μg) was used for cDNA synthesis using a QuantiTect Reverse transcription Kit (Qiagen, Hilden, German). Real-time PCR was performed using a SYBR green PCR kit (TOYOBO, Japan) in an ABI 7500 Sequence Detection System (Applied Biosystems, Sunnyvale, CA). The sequences of the primers used to amplify B7-H6 were 5'-TCACCAAGAGGCATTCCGACCT-3' (sense) and 5'-ACCACCTCACATCGGTACTCTC-3' (anti-sense). The housekeeping gene GAPDH was used as an internal control.

### Western blot analysis

Cytoplasmic proteins were prepared as previously described ^8^. Equal amounts of protein extract (30μg) were separated by SDS-PAGE and transferred to the PVDF membranes.Nonspecific binding sites were blocked with 5% nonfat milk. Blots were incubated with rabbit polyclonal to B7-H6 antibody (Catalog # ab138588, Abcam, USA) overnight at 4°C. The membranes were washed three times in phosphate-buffered saline containing 0.1% Tween-20 for 1 h at room temperature and were incubated with HRP-conjugated secondary antibody (Catalog # ab97051, Abcam, USA). The membranes were washed as described above. Proteins were visualized by Dura SuperSignal Substrate (Pierce, USA).

### Statistical Analysis

Data were expressed as the mean ± SD. The means between groups were analyzed using Kruskal-Wallis analysis of variance or the Mann-Whitney U test. The degree of correlation between variables was assessed using Spearman’s correlation. All statistical analyses were performed using SPSS v16.00 statistical analysis software (SPSS Inc., Chicago, IL). Differences were considered statistically significant if *P* < 0.05.

## Results

### Hepatic B7-H6 expression was enhanced in HBV-ACLF patients

**Fig 1:**
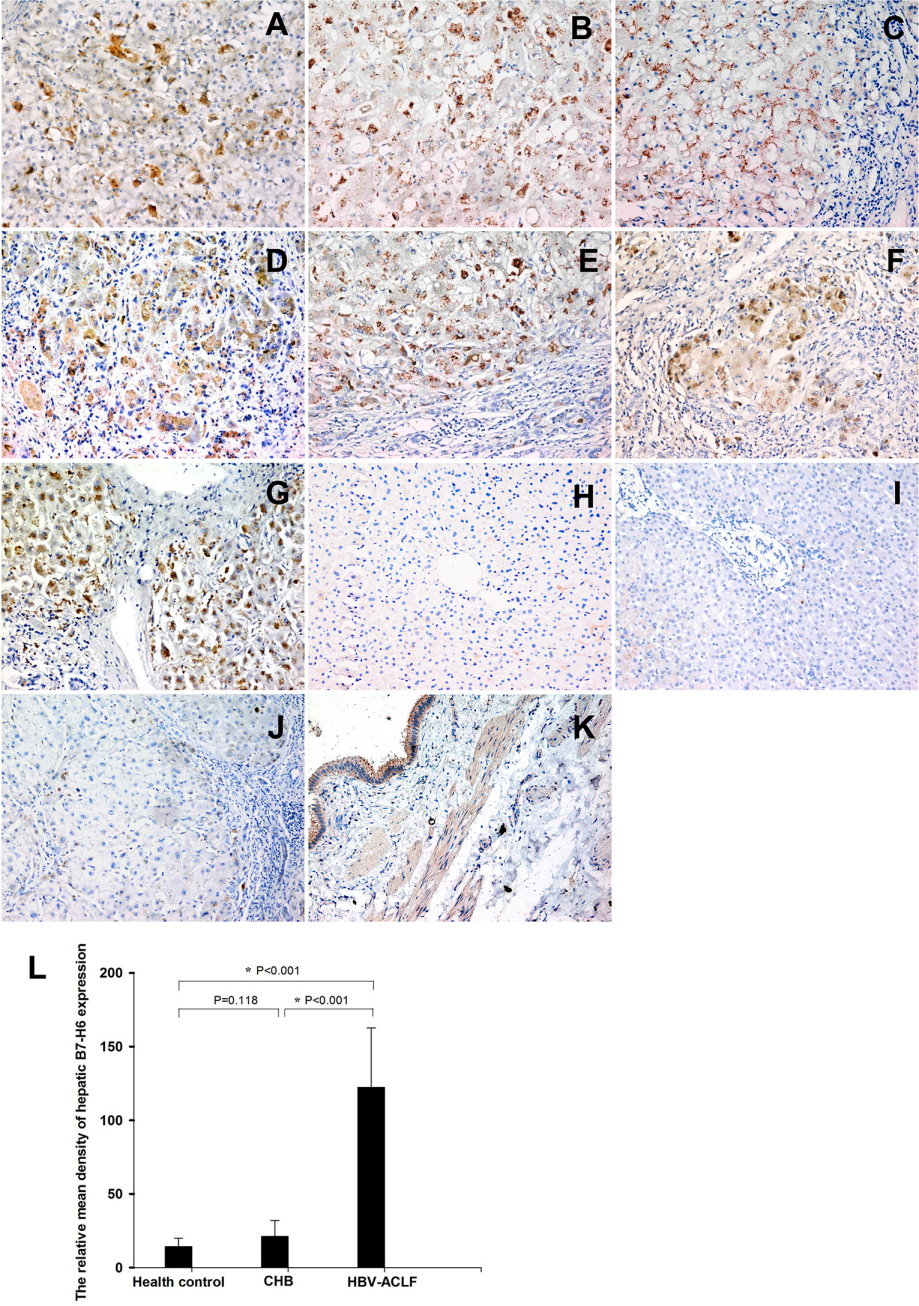
Enhanced expression of hepatic B7-H6 in HBV-ACLF patients. The detection of hepatic B7-H6 expression in patients with HBV-ACLF (Fig 1, A–G, original magnification 200x), mild CHB (Fig H, original magnification 200x), healthy controls (Fig 1I, original magnification 200x) was performed by immunohistochemical staining. B7-H6 staining with rabbit serum as first antibody was used as experimental negative controls (Fig 1J, original magnification 200x). According to the manufacturer's instructions, B7-H6 staining in human gallbladder tissue was used as the positive control (Fig 1K, original magnification 200x). Relative mean density analysis indicated that hepatic B7-H6 expression was significantly higher in HBV-ACLF patients than in mild CHB patients or in healthy controls (*P* < 0.05).

We analyzed the expression of B7-H6, a NKp30 ligand, in liver tissues from twenty patients with HBV-ACLF (Fig 1A–1G), twenty patients with mild CHB (Fig 1H), and five healthy controls (Fig 1I) by immunohistochemistry. Relative mean density analysis indicated that the incidence of hepatic B7-H6 expression was significantly higher in HBV-ACLF patients than in patients with mild CHB or in healthy controls (Fig 1J, *P* < 0.05). Immunohistochemical staining showed that the B7-H6 protein was mainly localized in cytoplasmic and at the cell membrane of hepatocytes which were undergoing apoptosis and necrosis. Normal hepatocytes do not express B7-H6.

### Hepatic B7-H6 expression positively correlates with liver injury severity in HBV-ACLF patients

**Fig 2:**
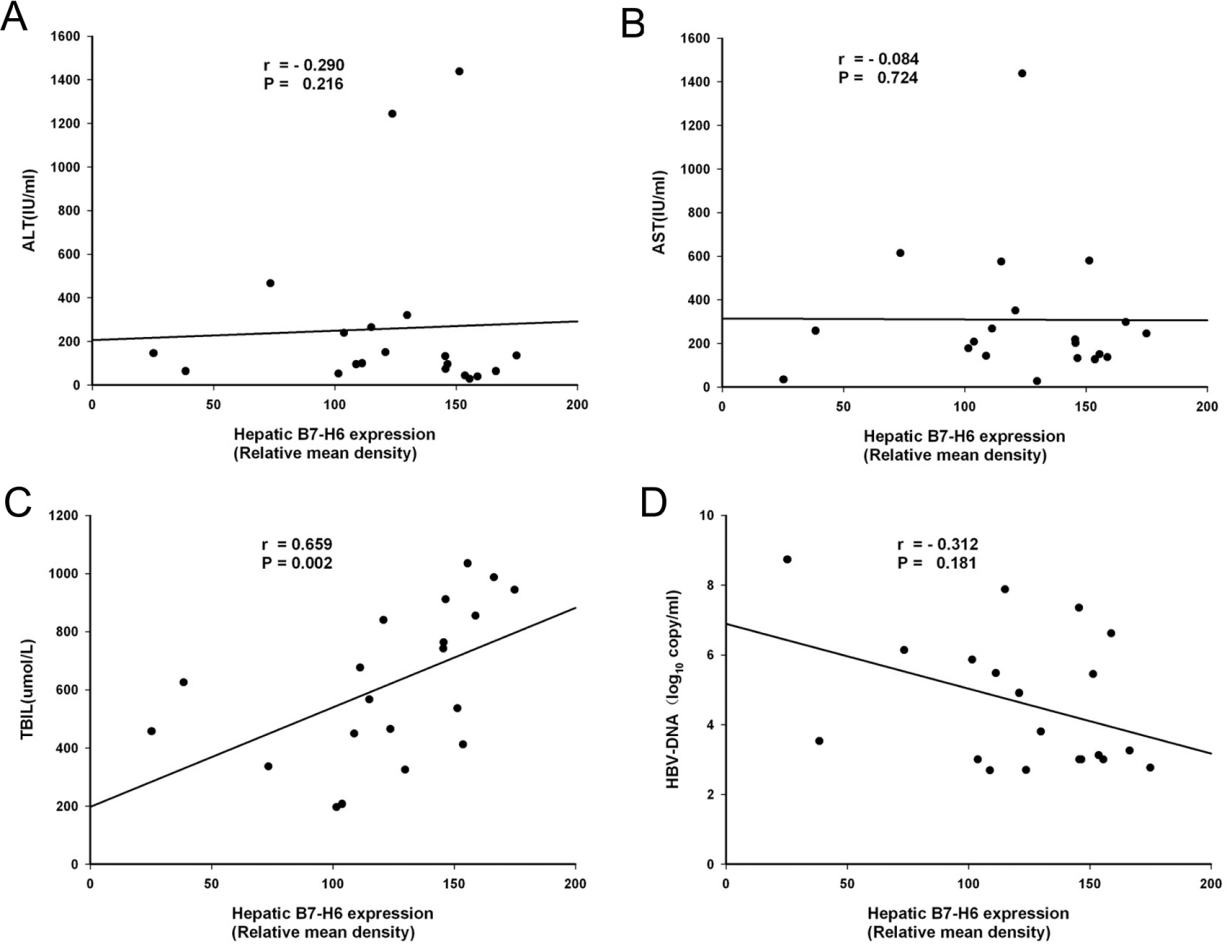
Hepatic B7-H6 expression was positively correlated with liver injury severity in HBV-ACLF patients. The relative mean density of hepatic B7-H6 staining was positively correlated with serum TBIL level in HBV-ALCF patients, with a correlation index of 0.659 (*P* < 0.05) (Fig 2C). The relative mean density of hepatic B7-H6 staining did not correlate with ALT, AST, or HBV levels (*P* > 0.05) (Fig 2, A, B and D).

The relative mean density of hepatic B7-H6 staining was positively correlated with liver injury severity, as evaluated by total bilirubin (TBIL) level, in HBV-ACLF patients (Fig 2C, *P* < 0.05), with a correlation index of 0.659. However, there was no correlation between hepatic B7-H6 staining density and alanine aminotransferase (ALT), aspartate aminotransferase (AST), hepatitis B virus DNA (HBV-DNA) levels (Fig 2A, 2B and 2D, *P* > 0.05). These results suggested that NKP30-B7H6 recognition may be involved in NK cell-mediated hepatocyte damage.

### Hepatic IL-32 expression was augmented in HBV-ACLF patients

**Fig 3:**
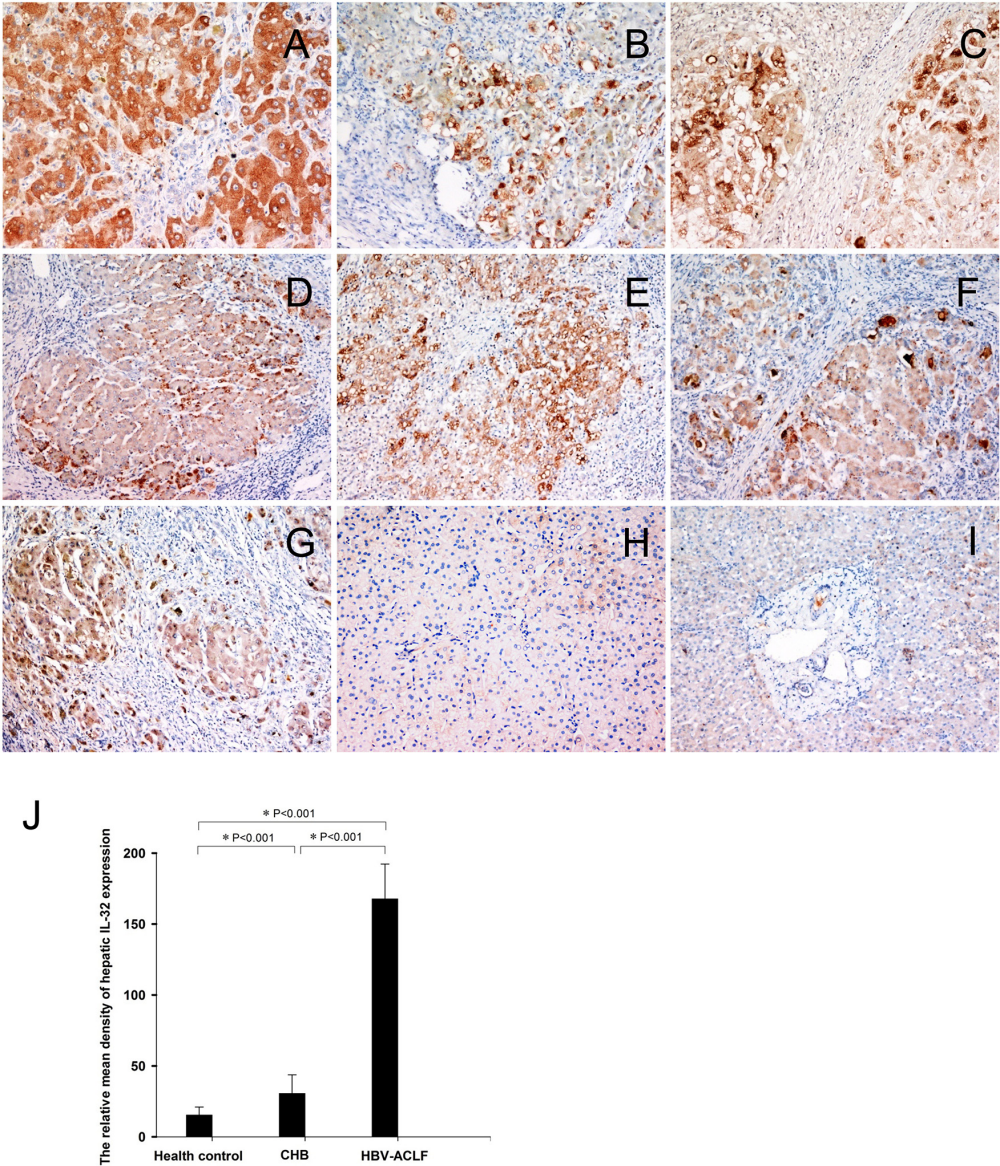
Up-regulated hepatic IL-32 expression in HBV-ACLF patients. IL-32 expression was detected in liver samples from patients with HBV-ACLF (Fig 3, A–G, original magniifcation 200x), mild CHB (Fig 3H, original magnification 200x), and healthy controls (Fig 3I, original magnification 200x) by immunohistochemical staining. Relative mean density analysis indicated that the hepatic B7-H6 expression in HBV-ACLF patients was significantly higher than that in mild CHB patients or healthy controls (*P* < 0.05) (Fig 3J).

We previously demonstrated that the expression of the proinflammatory cytokine IL-32 was correlated with the severity of liver inflammation and fibrosis in patients with chronic HBV infection [12]. To further investigate the role of IL-32 in immune cell-mediated hepatocyte damage in HBV-ACLF, we analyzed the hepatic IL-32 expression in twenty patients with HBV-ACLF (Fig 3A–3G), twenty patients with mild CHB (Fig 3H), and five healthy controls (Fig 3I). Image analysis revealed that the relative mean density of hepatic IL-32 staining in HBV-ACLF patients was significantly higher than that in mild CHB patients and health controls (Fig 3J, *P* < 0.05). Immunohistochemical staining showed that the IL-32 protein was expressed in nearly all hepatocytes and that some inflammatory cells infiltrated the liver in the cases of HBV-ACLF.

### Hepatic IL-32 expression is positively correlated with liver injury severity in HBV-ACLF

**Fig 4:**
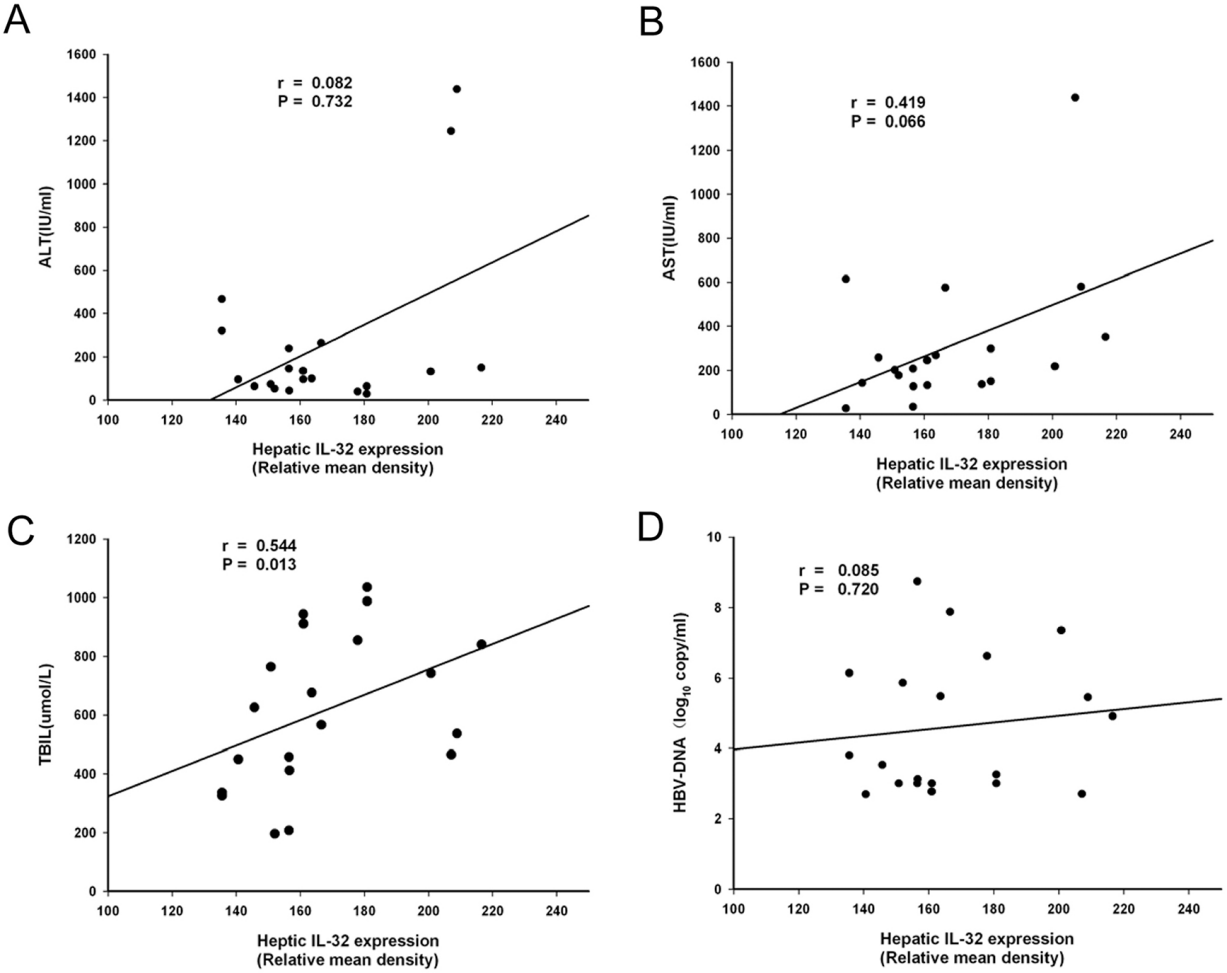
Hepatic IL-32 expression was positively correlated with liver injury severity in HBV-ACLF patients. The relative mean density of hepatic IL-32 staining was positively correlated with serum TBIL level in HBV-ALCF patients, with a correlation index of 0.544 (*P* < 0.05) (Fig 4C). The relative mean density of hepatic B7-H6 staining did not correlate with ALT, AST, or HBV levels (*P* > 0.05) (Fig 4, A, B and D).

The relative mean density of hepatic IL-32 staining was positively correlated with the severity of liver injury (as evaluated by TBIL level) in HBV-ACLF patients (Fig 4C, *P* < 0.05), with a correlation index of 0.544. However, there was no correlation between IL-32 staining density and alanine aminotransferase (ALT), aspartate aminotransferase (AST) or hepatitis B virus DNA (HBV-DNA) levels (Fig 4A, 4B and 4D, *P* > 0.05). These data strongly suggest that IL-32 plays an important role in hepatocyte damage in HBV-ACLF.

### Positive correlation between hepatic B7-H6 and IL-32 expression

**Fig 5:**
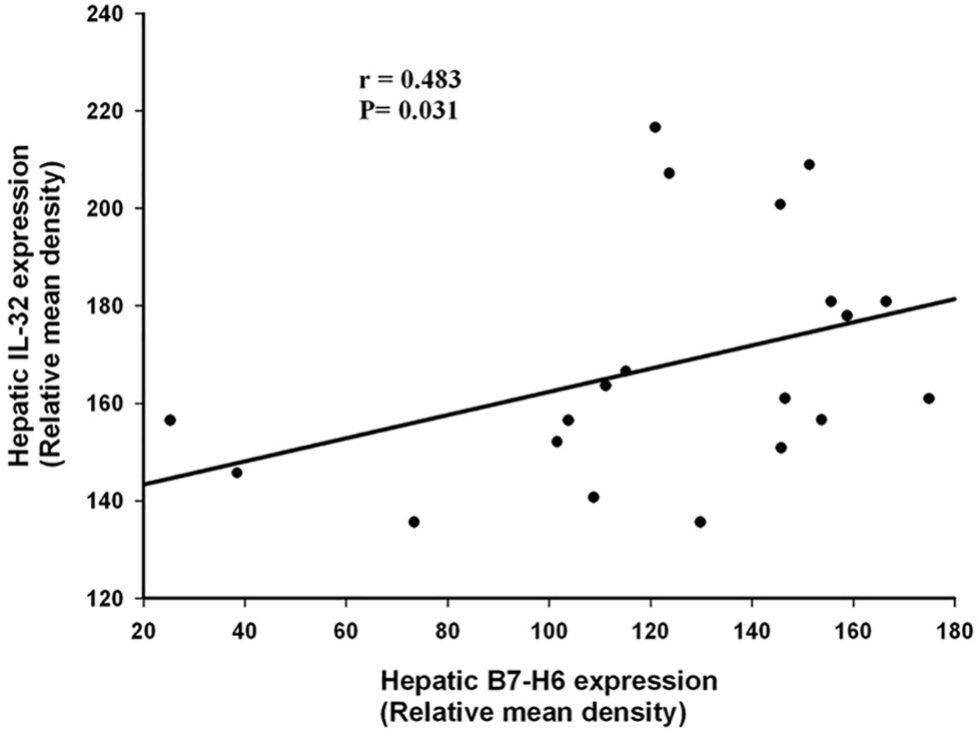
Positive correlation between hepatic B7-H6 and IL-32 Expression. The relative mean density of hepatic B7-H6 staining was positively correlated with the relative mean density of hepatic B7-H6 staining serum, with a correlation index of 0.483 (*P* < 0.05) (Fig 5).

We assessed the relationship between hepatic B7-H6 and IL-32 expression in HBV-ACLF patients and found that there was positive association between the relative mean density of hepatic B7-H6 and IL-32 staining (Fig 5, *P* < 0.05), with a correlation index of 0.483, which suggest IL-32 may be involved in the NKP30-B7H6 specific recognition in the NK-cell mediated liver injury in HBV-ACLF.

### Expressions of IL-32 in liver NK cells and T cells were enhanced in HBV-ACLF patients

To better confirm that intra-hepatic NK cells are an important the source of IL-32 in HBV-ACLF, we isolated the liver mononuclear cells from patients with HBV-ACLF or mild CHB and detected IL-32 expression in liver NK cells or T cells by the multicolor flow cytometric analysis. As depicted in S1 Fig, compared with mild CHB patients, the expressions of IL-32 in liver NK cells and T cells from patients with HBV-ACLF patients were both upregulated (39.40±4.80% vs. 3.36±1.24%, 40.52±7.33% vs. 5.87±1.92%, *P* < 0.05).

### NKP30-B7-H6 interaction contribute to the NK cell-mediated cytotoxicity against hepatoma cells

**Fig 6:**
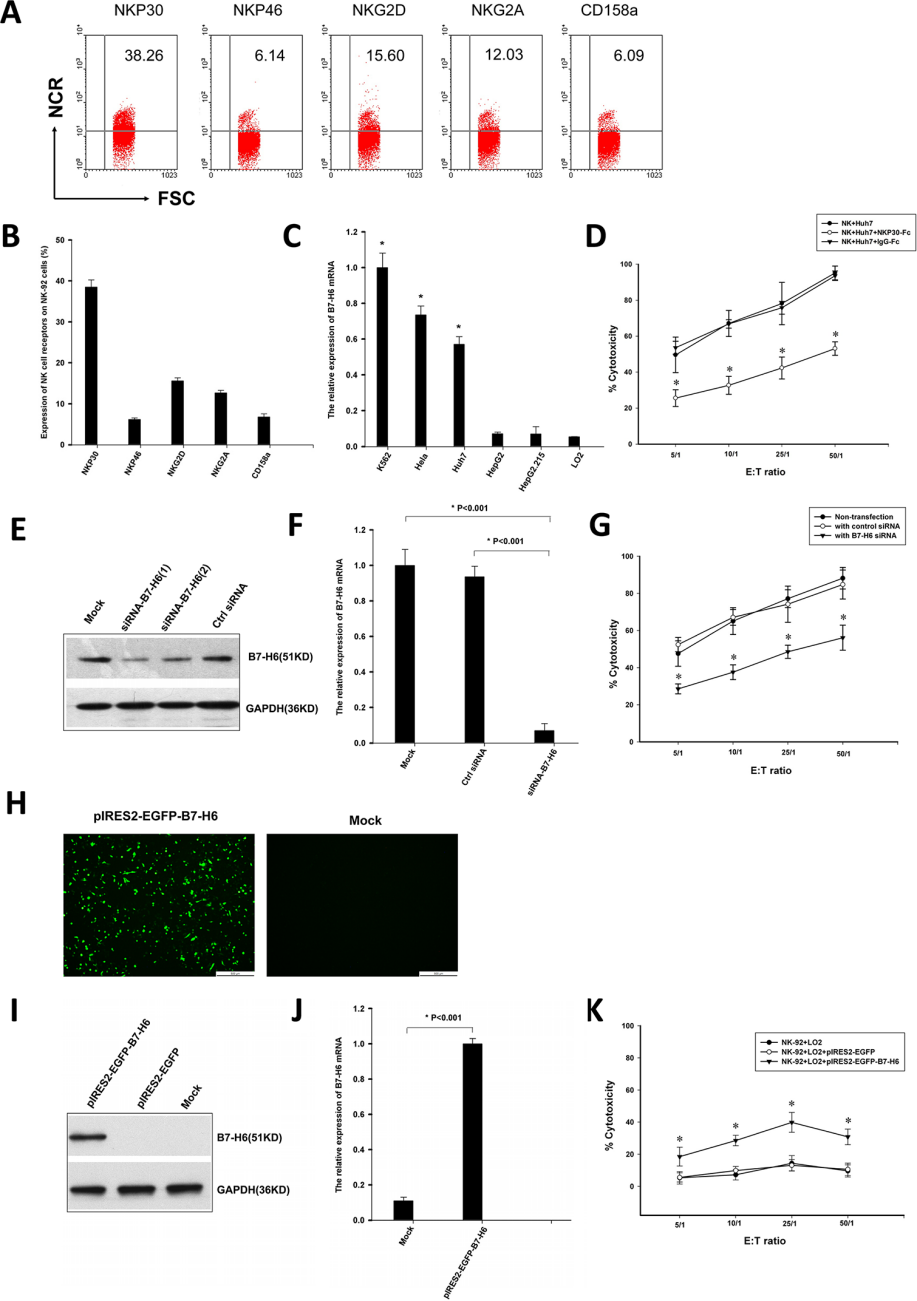
NKP30-B7-H6 interaction contributes to the NK cell-mediated hepatocyte toxicity. Cytotoxicity assays were performed to confirm the role of NKP30-B7H6 recognition in NK cell-mediated cytotoxicity against hepatoma cells and hepatocytes. NK-92 cells expressing high levels of NKp30 were used as effector cells. Hepatoma cell line Huh7 transfected with or no B7-H6 siRNA and LO2 cells transfected with or no pIRES2-EGFP-B7-H6 plasmid were used target cells. (A) representative FACS analysis of NK cells receptors on NK-92 cells. (B) A statistic analyses of the frequencies of NK-92 cells expressing NK cell receptors. Results are the mean ± SD. (C) B7-H6 mRNA expression in Huh7, HepG2, HepG2.215, LO2, K562, and HeLa cells. Results are the mean ± SD. *P < 0.05 as comparisons with HepG2, HepG2.215 and LO2 groups. (D) Soluble NKp30-Fc inhibited the killing of NK-92 cells against Huh7 cells. A 4-h cytoxicity assay was performed in the presence or absence of 2ug/ml NKp30-Fc. Transfection of Huh7 cells with B7-H6 siRNA resulted in silencing of B7-H6 gene as assessed by quantitative real time PCR (E) and western blotting (F). Results are the mean ± SD. *P* values are shown. (G) Effect of B7-H6 gene knockdown on the cytotoxicity of NK-92. Huh7 cells were transfected with non-targeting control siRNA or B7-H6 siRNA by lipofectamine and then incubated for 48 hr, thereafter co-cultured with NK-92 cells at different effector/target ratios for 4 h. The cytotoxicity of NK-92 cell was evaluated by the CCK8 assay. Results are the mean ± SD. *P < 0.05 as comparisons with non-transfection and control siRNA groups. The recombinant pIRES2-EGFP-B7-H6 plasmid was transfected into LO2 cells, B7-H6 expression was detected under fluorescence microscope (H) and confirmed by quantitative real time PCR (I) and western blotting (J). Results are the mean ± SD. *P* values are shown. (K) Effect of over-expression of the B7-H6 gene on the cytotoxicity of NK-92 cells. LO2 cells were transfected with control pIRES2-EGFP or pIRES2-EGFP-B7-H6 plasmid by lipofectamine and then incubated for 48 h, thereafter co-cultured with NK-92 cells at different effector/target ratios for 4 h. The cytotoxicity of NK-92 cell was evaluated by the CCK8 assay. Results are the mean ± SD. *P < 0.05 as comparisons with non-transfection and control pIRES2-EGFP plasmid groups.

The ratio of human NK-92 cell expresses NKP30, NKP46, NKG2A, NKG2D and CD158a is 38.5%±1.7%, 6.2%±0.3%, 12.7±0.6%, 15.6%±0.7% and 6.8%±0.7%, respectively. (Fig 6A and 6B). B7-H6 mRNA expression was detected in several cell lines, including Huh7, HepG2, HepG2.215, LO2, K562, and HeLa cells. The B7-H6 mRNA expression levels in Huh7, K562 and HeLa cell lines were higher than those observed in HepG2, HepG2.215 and LO2 cell lines (Fig 6C, *P* < 0.05). Cytotoxicity assays were performed to confirm the role of NKP30-B7H6 recognition in NK cell-mediated cytotoxicity against hepatoma cells and hepatocytes. NK-92 cells expressing high levels of NKp30 were used as effector cells, hepatoma cell line Huh7 transfected with or no B7-H6 siRNA and LO2 cells transfected with or no pIRES2-EGFP-B7-H6 plasmid were used target cells. The results showed that NK-92 cells are highly capable of killing the high B7-H6 expressing Huh7 cells, and the cytotoxic effect was markedly inhibited by soluble NKP30-Fc protein (Fig 6D). siRNA-mediated B7-H6 knockdown remarkably impaired NKp30-dependent killing of NK-92 cells (Fig 6G). The cytotoxic effect of NK-92 cells on LO2 cells can be enhanced by transfecting LO2 cells with pIRES2-EGFP-B7-H6 plasmid (Fig 6K). These results suggest an important involvement of NKP30-B7-H6 interaction in the NK cell-mediated cytotoxicity against hepatoma cells and hepatocytes.

### NKP30-B7-H6 interaction enhanced IL-32 expression and induced hepatoma cells apoptosis

**Fig 7:**
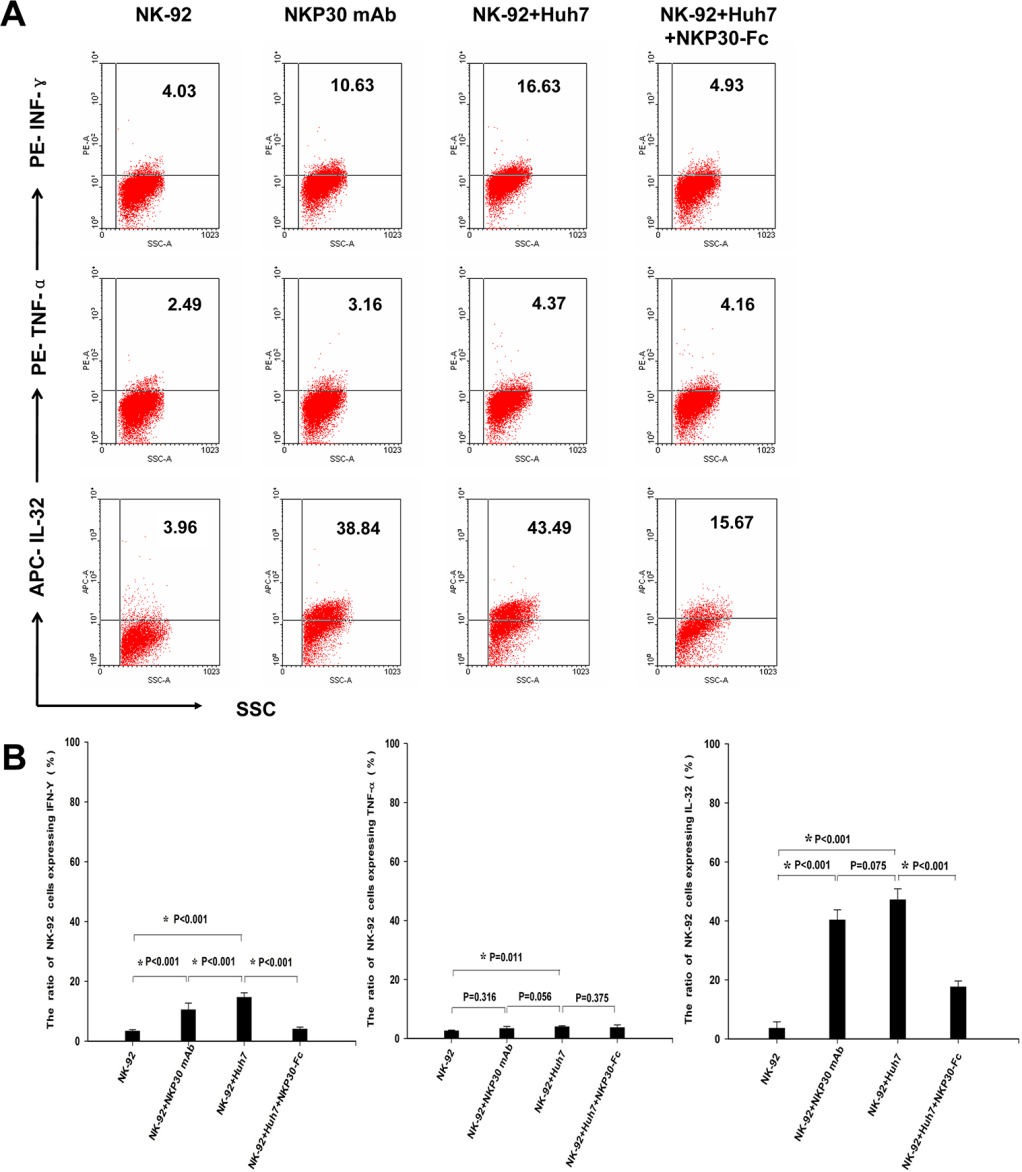
NKP30-B7-H6 interaction enhanced IL-32 expression. The expression of IL-32 in NK-92 cells was detected upon stimulation with an anti-NKP30 antibody or co-culture with Huh7 cells at an E: T ratio of 25:1 in either the presence or absence of NKP30-Fc protein. (A) A representative intracellular cytokine staining assay of the expression of IL-32 in NK-92 cells. (B) A statistical analyses of the expression of IL-32 in NK-92 cells. The results are presented as the mean ± SD of three independent experiments. * *P* < 0.05 as comparisons with other groups.

**Fig 8:**
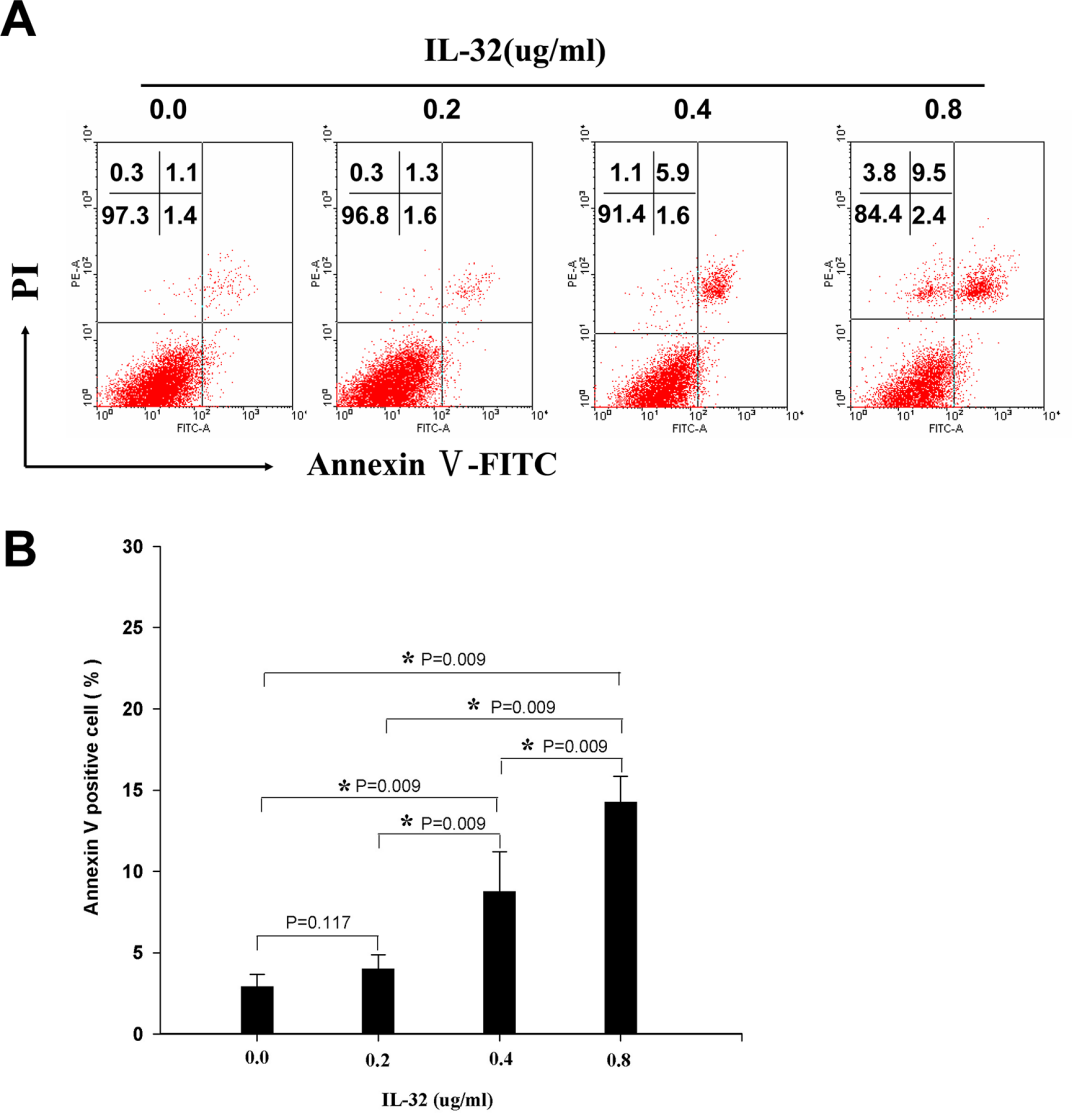
Effect of IL-32 on apoptosis induction among Huh7 cells. Huh7 cells were treated with various concentration of recombinant Human IL-32γ (0–0.4μg/ml, R&D Systems) for 48h followed by FITC–Annexin V/PI flow cytometry analyses. (A) A representative FITC–Annexin V/PI assay of Huh7 cells. (B) A statistical analyses of the percentage of annexin V-positive Huh7 cells. The results are presented as the mean ± SD of three independent experiments.

To further investigate the correlation of NKP30-B7-H6 interaction and the expression of IL-32, we detected the expression of IL-32 by NK-92 cells by the intracellular cytokine staining assay. It was interesting that NK-92 cells exhibited markedly elevated IL-32 expression when stimulated with an anti-NKP30 antibody or co-cultured with Huh7 cells. Treatment with NKP30-Fc protein remarkably inhibited the IL-32 expression in NK-92 cells co-cultured with Huh7 cells (Fig 7A and 7B). Furthermore, IL-32 induced the apoptosis of Huh7 cells in a dose-dependent manner (Fig 8A and 8B).Taken together, these data demonstrate that the NKP30-B7-H6 interaction induced IL-32 expression and enhanced NK cell-mediated cytotoxicity against hepatoma cells.

## Discussion

It has been confirmed that B7-H6 is a tumor cell ligand that activates the natural killer cell receptor NKp30 in humans, and B7-H6-NKP30 recognition may trigger the anti-tumor effect of NK cells [13, 14]. Our previous study demonstrated the accumulation of hepatic NK cells and the up-regulation of natural cytotoxicity receptors (NKP30 and NKP46) on peripheral NK cells in HBV-ACLF patients [10]. The role of B7-H6-NKP30 in NK cell-mediated liver injury in the context of HBV infection remains unclear. Thus, we analyzed hepatic B7-H6 expression in HBV-ACLF patients. B7-H6 has been previously shown to be the tumor cell ligand for NKP30 in humans; this protein is not detected in normal human tissues but is selectively expressed in a variety of human tumor cell lines, including T and B lymphomas, melanomas, and carcinomas [13]. We report here the first observation of markedly enhanced B7-H6 expression on HBV-infected hepatocytes in HBV-ACLF patients. The up-regulation of B7-H6 expression was positively correlated with liver injury severity in HBV-ACLF patients. These results showed that B7-H6, as a stress-induced self-molecule, may play a pivotal role in triggering the innate immune response in virus infection- and immune-mediated liver failure.

Interleukin-32 (IL-32) was previously called natural killer cell transcript 4 [15]. The main sources of IL-32 are natural killer cells, T cells, epithelial cells, and blood monocytes [16, 17]. IL-32 is an important proinflammatory cytokine that plays an important role in the innate and adaptive immune responses. IL-32 can induce the expression of IL-1, TNF-α and IL-6 by monocytes and macrophages [18, 19]. In addition, IL-32 can enhance the cytotoxic effect of natural killer cells against cancer cells [20, 21]. Our previous study demonstrated that HBX can induced IL-32 expression through activation of NF-κB in Huh7 cells[22]. Hepatic IL-32 expression was correlated with the severity of liver inflamm ation/fibrosis in patients with chronic HBV infection [12]. In the present study, we confirmed that hepatic IL-32 expression was augmented in HBV-ACLF patients, which was positively correlated with liver injury severity. The liver parenchymal cells, liver NK cells and T cell expressed high level of IL-32. In intro cytotoxicity assay demonstrated that NKP30-B7-H6 interaction enhanced IL-32 expression and induced hepatoma cells apoptosis.

Emerging evidence shows that activated hepatic plasmacytoid dendritic cells (pDCs) and hepatic NK cells are accumulated in large numbers in the livers of the HBV-ACLF patients [23]. Cross-talk between NK and with DCs through the interaction of the NKp30 receptor with its ligands induces DC maturation and NK cell activation [24]. Additionally, IL-32 can induce the maturation and activation of immature DCs and lead to enhanced Th1 and Th17 responses [25]. Based on these findings, we speculated that the IL-32 produced by hepatic NK cells and T cells induced the maturation and activation of DCs, which in turn promote the proliferation and activation of NK cells and lead to enhanced Th1, Th17 and NK cell responses. A strong cellular immune response and cytokine activation aggravated the inflammatory response and induced hepatocyte damage in HBV-ACLF.

It should be noted that we used an artificial system (hepatoma cells and an NK-cell line) in in vitro cytotocicity assay to investigate the role of NKP30-B7-H6 recognition in HBV-ACLF. Although neither primary hepatocytes nor primary intrahepatic NK cells have been used and that results from the in vitro system might not be directly transferable, the findings are clear and provide a molecular targets for further clinical investigation.

In summary, this study investigated the role of NKP30-B7-H6 interaction in NK cell-mediated hepatocyte damage in HBV-ACLF. NKP30-B7-H6 interaction aggravated hepatocyte damage through the up-regulation of IL-32 expression in HBV-ACLF. These results provide new insights into the role of the specific recognition of natural cytotoxicity receptors and ligands in the pathogenesis of HBV-ACLF.

## Supporting Information

S1 Fig Enhanced expression of IL-32 in liver NK cells and T cells from patients with HBV-ACLF. The expresion of IL-32 in liver NK cells and T cells from patients with HBV-ACLF or mild CHB was detected by the flow cytometric analysis. (A) A representative of the expression of IL-32 in liver NK cells and T cells. (B) A statistic analyze of the percentage of liver NK cells and T cells expressing IL-32. Results are the mean ± SD. *P* values are shown.
(TIF)

S1 Table Characteristics of the patients (Flow cytometric analysis). (DOCX)

## References

[1] Marsden PA, Ning Q, Fung LS, Luo X, Chen Y, Mendicino M, et al The Fgl2/fibroleukin prothrombinase contributes to immunologically mediated thrombosis in experimental and human viral hepatitis. J Clin Invest. 2003; 112: 58–66. 12840059 10.1172/JCI18114 PMC162293

[2] Liu M, Chan CW, McGilvray I, Ning Q, Levy GA. Fulminant viral hepatitis: molecular and cellular basis, and clinical implications. Expert Rex Mol Med. 2001; 3: 1–19. 10.1017/S1462399401002812 14987358

[3] Sarin SK, Kumar A, Almeida JA, Chawla YK, Fan ST, Garg H, et al Acute-on-chronic liver failure: consensus recommendations of the Asian Pacific Association for the study of the liver (APASL). Hepatol Int. 2009; 3: 269–282. 10.1007/s12072-008-9106-x 19669378 PMC2712314

[4] Liu Q, Liu Z, Wang T, Wang Q, Shi X, Dao W. Characteristics of acute and sub-acute liver failure in China: nomination, classification and interval. J Gastroenterol Hepatol. 2007; 22: 2101–2106. 18031366 10.1111/j.1440-1746.2006.04362.x

[5] Custer B, Sullivan SD, Hazlet TK, Iloeje U, Veenstra DL, Kowdley KV. Global epidemiology of hepatitis B virus. J Clin Gastroenterol. 2004; 38: S158–S168. 15602165 10.1097/00004836-200411003-00008

[6] Liang TJ. Hepatitis B: the virus and disease. Hepatology. 2009; 49: S13–S21 10.1002/hep.22881 19399811 PMC2809016

[7] Wu Z, Han M, Chen T, Yan W, Ning Q. Acute liver failure: mechanisms of immune-mediated liver injury. Liver Int. 2010; 30: 782–794. 10.1111/j.1478-3231.2010.02262.x 20492514

[8] Roth E, Pircher H. IFN-gamma promotes Fas ligand- and perforin-mediated liver cell destruction by cytotoxic CD8 T cells. J Immunol. 2004; 172: 1588–1594. 14734739 10.4049/jimmunol.172.3.1588

[9] Maini MK, Boni C, Lee CK, Larrubia JR, Reignat S, Ogg GS, et al The role of virus-specific CD8 (+) cells in liver damage and viral control during persistent hepatitis B virus infection. J Exp Med. 2000; 191: 1269–1280. 10770795 10.1084/jem.191.8.1269 PMC2193131

[10] Zou Y, Chen T, Han M, Wang H, Yan W, Song G, et al Increased killing of liver NK cells by Fas/Fas ligand and NKG2D/NKG2D ligand contributes to hepatocyte necrosis in virus-induced liver failure. J Immunol. 2010; 18: 466–475. 10.4049/jimmunol.0900687 19949088

[11] Sarin SK, Kumar A, Almeida JA, Chawla YK, Fan ST, Garg H, et al Acute-on-chronic Liver failure: consensus recommendations of the Asian Pacific Association for The study of the liver (APASL). Hepatol Int. 2009; 3: 269–282. 10.1007/s12072-008-9106-x 19669378 PMC2712314

[12] Xu Q, Pan X, Shu X, Cao H, Li X, Zhang K, et al Increased interleukin-32 expression in chronic hepatitis B virus-infected liver. J Infect. 2012; 65: 336–342. 10.1016/j.jinf.2012.05.009 22687868

[13] Brandt CS, Baratin M, Yi EC, Kennedy J, Gao Z, Fox B. The B7 family member B7-H6 is a tumor cell ligand for the activating natural killer cell receptor NKp30 in humans. J Exp Med. 2009; 206: 1495–1503. 10.1084/jem.20090681 19528259 PMC2715080

[14] Li Y, Wang Q, Mariuzza RA. Structure of the human activating natural cytotoxicity receptor NKp30 bound to its tumor cell ligand B7-H6. J Exp Med. 2011, 208: 703–714. 10.1084/jem.20102548 21422170 PMC3135353

[15] Dahl CA, Schall RP, He HL, Cairns JS. Identification of a novel gene expressed in activated natural killer cells and T cells. J Immunol. 1992; 148: 597–603. 1729377

[16] Kim SH, Han SY, Azam T, Yoon DY, Dinarello CA. Interleukin-32: a cytokine and inducer of TNFα.Immunity. 2005; 22: 131–142. 15664165 10.1016/j.immuni.2004.12.003

[17] Shoda H, Fujio K, Yamaguchi Y, Okamoto A, Sawada T, Kochi Y, et al Interactions between IL-32 and tumor necrosis factor α contribute to the exacerbation of immune-inflammatory diseases. Arthritis Res Ther. 2006; 8: R166 17078892 10.1186/ar2074 PMC1794509

[18] Nold MF, Nold-Petry CA, Pott GB, Zepp JA, Saavedra MT, Kim SH, et al Endogenous IL-32 controls cytokine and HIV-1production. J Immunol. 2008; 181: 557–565. 18566422 10.4049/jimmunol.181.1.557

[19] Dinarello CA, Kim SH. IL-32, a novel cytokine with a possible role in disease. Ann Rheum Dis. 2006; 65: iii61–iii64. 17038476 10.1136/ard.2006.058511 PMC1798381

[20] Cheon S, Lee JH, Park S, Bang SI, Lee WJ, Yoon DY, et al Overexpression of IL-32 alpha increases natural killer cell-mediated killing through up-regulation of Fas and UL16-binding protein 2 (ULBP2) expression in human chronic myeloid leukemia cells. J Biol Chen. 2011; 286: 12049–12055. 10.1074/jbc.M110.159756 PMC3069408 21321117

[21] Park MH, Song MJ, Cho MC, Moon DC, Yoon, Han SB, et al Interleukin-32 enhances cytotoxic effect of natural killer cells to cancer cells via activation of death receptor 3. Immunology. 2012; 135: 63–72. 10.1111/j.1365-2567.2011.03513.x 22043900 PMC3246653

[22] Pan X, Cao H, Lu J, Shu X, Xiong X, Hong X, et al Interleukin-32 expression induced by hepatitis B virus protein X is mediated through activation of NF-κB. Mol Immunol. 2011; 48:1573–1577. 10.1016/j.molimm.2011.03.012 21481941

[23] Zhang Z, Zou ZS, Fu JL, Cai L, Jin L, Liu YJ, et al Severe dendritic cell perturbation is actively involved in the pathogenesis of acute-on-chronic hepatitis B liver failure. J Hepatol. 2008; 49: 396–406. 10.1016/j.jhep.2008.05.017 18644645

[24] Ferlazzo G, Tsang ML, Moretta L, Melioli G, Steinman RM, Munz C. Human dendritic cells activate resting natural killer (NK) cells and are recognized via the NKp30 receptor by activated NK cells. J Exp Med. 2002; 195: 343–351. 11828009 10.1084/jem.20011149 PMC2193591

[25] Jung MY, Son MH, Kim SH, Cho D, Kim TS. IL-32gamma induces the maturation of dendritic cells with Th1- and Th17-polarizing ability through enhanced IL-12 and IL-6 production. J Immunol. 2011; 186: 684–659. 10.4049/jimmunol.1003996 21551364

