# Supplementary figures and images for: NKP30-B7-H6 Interaction Aggravates Hepatocyte Damage through Up-Regulation of Interleukin-32 Expression in Hepatitis B Virus-Related Acute-On-Chronic Liver Failure
Source: PLoS One. 2015 Aug 4;10(8):e0134568. doi: 10.1371/journal.pone.0134568 (PMC4524618; doi:10.1371/journal.pone.0134568)

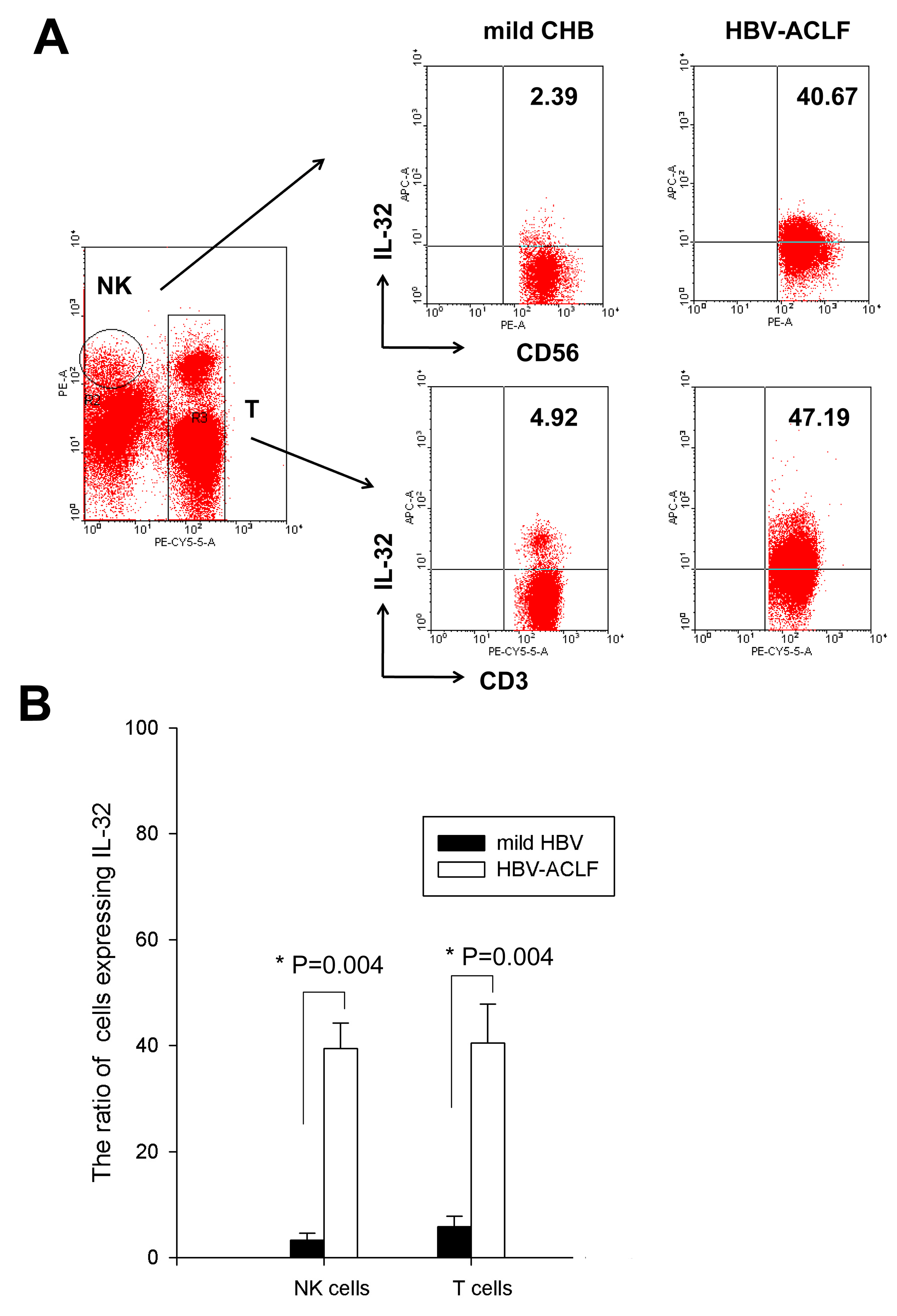

Supplement: S1 Fig — The expresion of IL-32 in liver NK cells and T cells from patients with HBV-ACLF or mild CHB was detected by the flow cytometric analysis. (A) A representative of the expression of IL-32 in liver NK cells and T cells. (B) A statistic analyze of the percentage of liver NK cells and T cells expressing IL-32. Results are the mean ± SD. P values are shown. (TIF) [file pone.0134568.s001.tif]
