# Supplementary material for: NKP30-B7-H6 Interaction Aggravates Hepatocyte Damage through Up-Regulation of Interleukin-32 Expression in Hepatitis B Virus-Related Acute-On-Chronic Liver Failure
Source: PLoS One. 2015 Aug 4;10(8):e0134568. doi: 10.1371/journal.pone.0134568 (PMC4524618; doi:10.1371/journal.pone.0134568)
Supplement: S1 Table — (DOCX) [file pone.0134568.s002.docx]

**S1 Table . Characteristics of the patients (Flow cytometric analysis)**

| **Characteristic** | **Mild CHB** | **HBV-ACLF** |
| --- | --- | --- |
| Number of patients | 10 | 10 |
| Age (years) | 27.5(23.5-33.8) | 40(36.0-44.0) |
| Male gender (n%) | 6(60.0%) | 8(80%) |
| HBsAg+ (%) | 100 | 80 |
| HBeAg+ (%) | 50 | 30 |
| Anti-HBcAg+ (%) | 100 | 100 |
| HBV DNA log_10_ (copies/ml)* | 7.7(7.2-8.1) | 3.4(3.0-5.6) |
| Serum ALT (IU/L) | 99.5(34.3-141.5) | 83 (44.5-133.4) |
| Serum total bilirubin (μmol/L)* | 17.4(14.8-18.8) | 532.5(397.1.3-623.2) |
| Prothronbinase time (PT)* | 12.0(11.6-12.6) | 34.9(27.9-39.1) |
| Prothrombinase activity (PTA)* | 98.5(91.5-104.5) | 25.0(20.8-27.9) |

* Median ( interquartile range)
